# Comparison of Oncologic Outcomes between Radical Hysterectomy and Primary Concurrent Chemoradiotherapy in Women with Bulky IB and IIA Cervical Cancer under Risk Stratification

**DOI:** 10.3390/cancers15113034

**Published:** 2023-06-02

**Authors:** Chung-Shih Chen, Eng-Yen Huang

**Affiliations:** 1 Departments of Radiation Oncology, Kaohsiung Chang Gung Memorial Hospital, Chang Gung University College of Medicine, Kaohsiung City 833, Taiwan; u780829@cgmh.org.tw; 2 School of Medicine, College of Medicine, National Sun Yat-Sen University, Kaohsiung City 804, Taiwan

**Keywords:** bulky early-stage cervical cancer, radical hysterectomy, concurrent chemoradiation

## Abstract

**Simple Summary:**

Bulky IB and IIA cervical cancer portends a worse prognosis than smaller tumors. Although treatment modalities include surgery or radiation (RT)-based treatment, there is no optimal treatment choice for these patients that is indicated by recurrence risk. The aim of our study was to compare the oncologic outcome between radical hysterectomy (RH) and concurrent chemoradiation (CCRT) under risk stratification according to tumor markers and histology. We defined patients with squamous cell carcinoma (SCC) with elevated carcinoembryonic antigen (CEA) ≥ 10 ng/mL, adenocarcinoma, or adenosquamous carcinoma as the high-risk group. We found that RH provided better locoregional control and relapse-free survival in the high-risk group. Meanwhile, there was a similar oncologic outcome regardless of treatment modality in patients without these risk factors. We suggest that surgery should be considered first for patients with these high-risk features of poor RT response.

**Abstract:**

Purpose: To stratify patients according to tumor marker and histology and compare the survival outcome between radical hysterectomy (RH) and primary concurrent chemoradiotherapy (CCRT) in bulky IB and IIA cervical cancer. Methods: A total of 442 patients with cervical cancer were enrolled in the Chang Gung Research Database from January 2002 to December 2017. Patients with squamous cell carcinoma (SCC) and carcinoembryonic antigen (CEA) ≥10 ng/mL, adenocarcinoma (AC), or adenosquamous carcinoma (ASC) were stratified into the high-risk (HR) group. The others were classified into the low-risk (LR) group. We compared oncology outcomes between RH and CCRT in each group. Results: In the LR group, 5-year overall survival (OS) and recurrence-free survival (RFS) were 85.9% vs. 85.4% (*p* = 0.315) and 83.6% vs. 82.5% (*p* = 0.558) in women treated with RH (*n* = 99) vs. CCRT (*n* = 179), respectively. In the HR group, the 5-year OS and RFS were 83.2% vs. 73.3% (*p* = 0.164) and 75.2% vs. 59.6% (*p* < 0.036) in patients treated with RH (*n* = 128) vs. CCRT (*n* = 36), respectively. Regarding recurrence, locoregional recurrence (LRR) (8.1% vs. 8.6%, *p* = 0.812) and distant metastases (DM) (17.8% vs. 21%, *p* = 0.609) were similar between RH and CCRT in the LR group. However, lower LRR (11.6% vs. 26.3%, *p* = 0.023) but equivalent DM (17.8% vs. 21%, *p* = 0.609) were found for women undergoing RH compared with CCRT in the HR group. Conclusions: There were similar survival and recurrence rates between both treatment modalities in low-risk patients. Meanwhile, primary surgery with or without adjuvant radiation provides better RFS and local control in women with high-risk features. Further prospective studies are needed to confirm these findings.

## 1. Introduction

Cervical cancer is the fourth most common malignant tumor and the fourth leading cause of death among women worldwide. In 2020, there were an estimated 604,000 new cases and 342,000 deaths worldwide [1]. Although early-stage cervical cancer portends a favorable prognosis, a large tumor size (>4 cm) is associated with worse survival and more local pelvic recurrence than a smaller tumor, regardless of treatment modality [2,3,4]. For early bulky tumors, treatment options include radical surgery (radical hysterectomy and lymphadenectomy) with or without adjuvant therapy, primary radiation, or concurrent chemoradiation. However, the optimal treatment is still under debate.

A previous randomized study [3] showed equivalent effectiveness by either primary surgery or radiotherapy, whereas two Surveillance, Epidemiology, and End Results (SEER) [5,6] studies revealed that surgery improved survival. Bansal et al. [5] reported that radical hysterectomy was associated with a 49% reduction in the mortality rate for 4–6 cm tumors but equivalent survival for >6 cm tumors compared with primary radiation. Moreover, radical hysterectomy with tailored adjuvant therapy has been suggested because it is the most cost-effective strategy in early bulky cervical tumors compared with primary chemoradiation [7,8]. Concerning the inconsistent conclusion from the above studies [3,5,6], there is a need to clarify suitable treatment modalities in this patient population.

In addition to tumor size, clinical prognostic factors, including tumor markers (SCC and CEA levels) and tumor histology (squamous cell carcinoma or adenocarcinoma), also have an impact on treatment response and survival [9,10,11]. The prognostic factors could be considered when determining better treatment options. However, there is a paucity of data on risk stratification for early-stage bulky tumors to guide treatment modality. Thus, the aim of this study was to assess the difference in clinical outcomes in patients who were grouped by their different prognoses between surgery and primary chemoradiation. We also sought to determine the suitable treatment modality for this patient population by risk stratification.

## 2. Materials and Methods

### 2.1. Patient Recruitment and Risk Classification

Our cohort study was reviewed and approved by the Institutional Review Board (IRB) of the Chang Gung Medical Foundation (IRB No. 202101639B0). From January 2002 to December 2017, patients diagnosed with cervical cancer were identified from the Chang Gung Research Database according to ICD 9 and 10. All patients with FIGO stages IB2 and IIA2, according to the 2009 FIGO staging system, were included. Patients with tumor sizes greater than 4 cm but recorded only as IB or IIA were also included. Regarding pathology, we enrolled patients with cervical squamous cell carcinoma (SCC), adenocarcinoma (AC), and adenosquamous carcinoma (ASC) based on ICD-O morphology codes. Patients with missing tumor marker data, including carcinoembryonic antigen (CEA) or SCC antigen (SCC-Ag) and pathology other than SCC/AC/ASC, were excluded. Because previous studies [10,11,12] have shown that pretreatment CEA levels and AC/ASC histology are associated with local failure and survival, we stratified the patients into high-risk and low-risk groups based on tumor markers and pathology. The high-risk group included patients with (1) SCC histology and CEA ≥ 10 ng/mL or (2) AC/ASC histology. The other patients without the above criteria belonged to the low-risk group.

### 2.2. Primary Treatment Modality

Each group was further divided into surgery and concurrent chemoradiation (CCRT) as the primary treatment. In the surgery group, the primary treatment included radical hysterectomy, which consisted of the removal of the uterus with the upper one-third to one-half of the vagina and parametrial tissues with pelvic and/or para-aortic lymphadenectomy. Patients undergoing simple hysterectomy were excluded. Adjuvant radiotherapy or chemoradiotherapy was performed for the intermediate-risk group and high-risk group, respectively, according to the pathologic risk factors. In the CCRT group, cisplatin-based chemotherapy was given during radiotherapy. The external beam radiation dose ranged from 39.6 to 70.2 Gy. External beam radiotherapy alone with a dose above 66.6 Gy was allowed because of difficulty with the insertion of the applicator for brachytherapy. The intracavitary brachytherapy dose in the patients who received brachytherapy ranged from 6 to 36 Gy. Sequential radiotherapy followed by chemotherapy or RT alone was excluded. In the low-risk group, 162 (90.5%) patients receiving CCRT completed brachytherapy, while 17 (9.5%) patients did not complete brachytherapy. In the high-risk group, 32 (88.9%) patients receiving CCRT completed brachytherapy, while four (11.1%) patients did not complete brachytherapy.

### 2.3. Statistical Analysis

The baseline patient characteristics included age, FIGO stage, tumor marker (SCC/CEA), and hemoglobin. To evaluate the differences in the baseline clinical factors, the continuous variables were compared between the surgery and CCRT treatment modalities in each risk group by the independent *t*-test, while the categorical variables were tested by either Pearson’s chi-square test or two-sided Fisher’s exact test. The evaluated clinical outcomes included locoregional recurrence (LRR), distant metastases (DM), overall survival (OS), and recurrence-free survival (RFS). Time to LRR was defined as the duration from the date of initial diagnosis to a local cervical tumor or regional nodal recurrence. Time to DM was determined by the interval between the date of initial diagnosis and the date of distant failure. OS was defined as the duration from the date of initial diagnosis to death or last follow-up. RFS was determined by the duration from the date of initial diagnosis to any event of the first recurrence (including LRR or DM), death, or last follow-up. The Kaplan-Meier method was performed for analysis, and the log-rank test was used to compare the differences in each treatment group. A Cox regression model was used to perform multivariate analysis. Variables included treatment modality, initial FIGO stage, hemoglobin, and histology differentiation. All statistical analyses were performed using SPSS v. 25.0 (IBM, Bloomington, IL, USA) with a two-tailed value of *p* < 0.05, indicating statistical significance.

## 3. Results

### 3.1. Study Population

**Figure 1:**
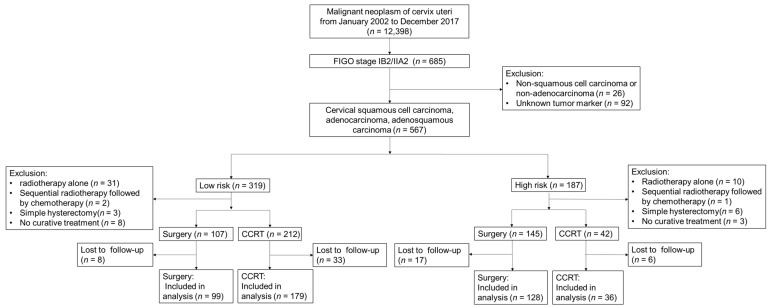
Flow chart of the cohort study. CCRT = concurrent chemoradiotherapy.

The cohort consisted of 685 patients diagnosed with either bulky IB2 or IIA2 cervical carcinoma according to 2009 FIGO staging in Chang Gung Medical Foundation, Taiwan, between January 2002 and December 2017. There were 567 patients with pathology of SCC, AC, or ASC after excluding other pathologies (*n* = 26) and unknown tumor markers (*n* = 92). After stratification by tumor marker and pathology, 319 patients were classified into the low-risk group, while 187 patients were classified into the high-risk group. In the low-risk group, exclusion criteria were applied, including radiotherapy alone (*n* = 31), sequential radiotherapy followed by chemotherapy (*n* = 2), simple hysterectomy (*n* = 3), and no curative treatment (*n* = 8). Forty-one patients were initially lost to follow-up after treatment. A total of 278 patients in the low-risk group were included for analysis, with 99 patients receiving surgery and 179 patients undergoing CCRT as initial treatment. In the high-risk group, patients undergoing radiotherapy alone (*n* = 10), sequential radiotherapy followed by chemotherapy (*n* = 1), simple hysterectomy (*n* = 6), and no curative treatment (*n* = 3) were excluded. Twenty-three patients were initially lost to follow-up after treatment. Finally, 164 patients in the high-risk group were included for analysis, with 128 patients receiving surgery and 36 patients undergoing CCRT as initial treatment. Figure 1 shows a flow chart of the study design from the cohort for the analysis.

### 3.2. Baseline Characteristics of the Patients

Table 1 shows the demographics and characteristics of the 278 and 164 patients in the low- and high-risk groups, respectively. More patients with FIGO stage IB2 received surgery in either the low-risk (85.9% vs. 67.6%, *p* = 0.001) or high-risk group (88.3% vs. 72.2%, *p* = 0.018), but there was no difference between the treatment modalities in the number of patients aged >45 years, pretreatment serum SCC-Ag, CEA concentration, or hemoglobin. In the high-risk group, there were 109 (66.5%) patients with AC, 30 (18.3%) patients with ASC, and 25 (15.2%) patients with SCC histology and CEA ≥ 10 ng/mL. In addition, 16 (64%) patients with SCC histology and CEA ≥ 10 ng/mL underwent CCRT, and 9 (36%) patients underwent surgery. On the other hand, 20 (14.4%) patients with AC/ASC underwent CCRT and 119 (85.6%) patients underwent surgery. The pathology of 20 (55.6%) patients receiving CCRT and 119 (93%) patients receiving surgery was AC/ASC in the high-risk group. In the surgery group of the entire cohort of 227 patients, 33 (14.5%) patients received adjuvant radiotherapy, and 93 (41%) patients received adjuvant chemoradiation. In the low-risk group, 25 (25.3%) patients receiving surgery underwent adjuvant RT, and 23 (23.2%) patients had adjuvant CRT. In the high-risk group, eight (6.3%) patients receiving surgery had undergone adjuvant RT, and 70 (54.7%) patients had adjuvant CRT. The adjuvant radiation dose ranged from 39.6 to 54 Gy. 

The clinical node-positive rate in the entire surgery group was 12.8%. In the surgery group, 14 (14.1%) and 15 (11.7%) patients had clinical nodal involvement in the low-risk and high-risk groups, respectively. Moreover, the pathologic node-positive rate in the entire surgery group was 26.9%. In the surgery group, 22 (22.2%) and 39 (30.5%) patients had pathologic nodal involvement in the low-risk and high-risk groups, respectively. On the other hand, the clinical node-positive rate in the entire CCRT group was 30.2%. In the CCRT group, 53 (29.6%) and 12 (33.3%) patients had clinical nodal involvement in the low-risk and high-risk groups, respectively.

Moreover, in the low-risk group, 29 (29.2%) patients underwent neoadjuvant chemotherapy (NACT) before surgery, while 6 (4.7%) patients underwent neoadjuvant chemotherapy in the high-risk group.

### 3.3. Oncologic Outcome and Multivariate Analysis

**Figure 2:**
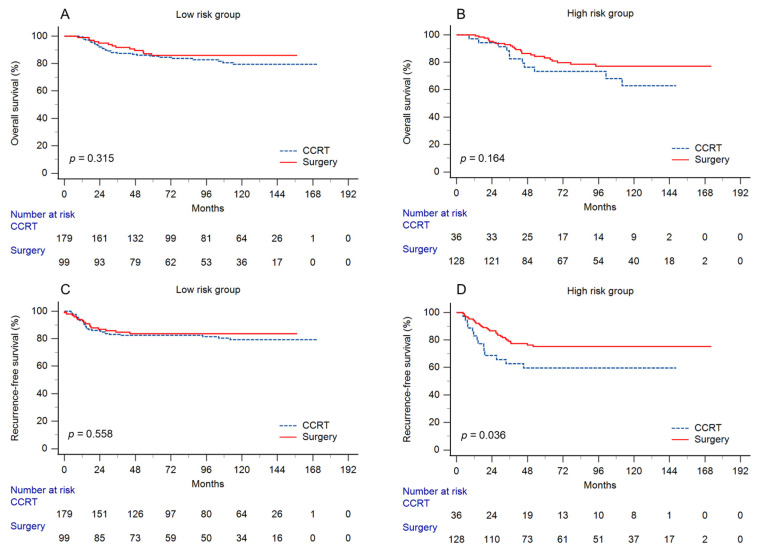
(**A**) Overall survival and (**C**) recurrence-free survival in the low-risk group. (**B**) Overall survival and (**D**) recurrence-free survival in the high-risk group by two treatment modalities. Patients treated with primary surgery had better recurrence-free survival than those treated with concurrent chemoradiation in the high-risk group.

**Figure 3:**
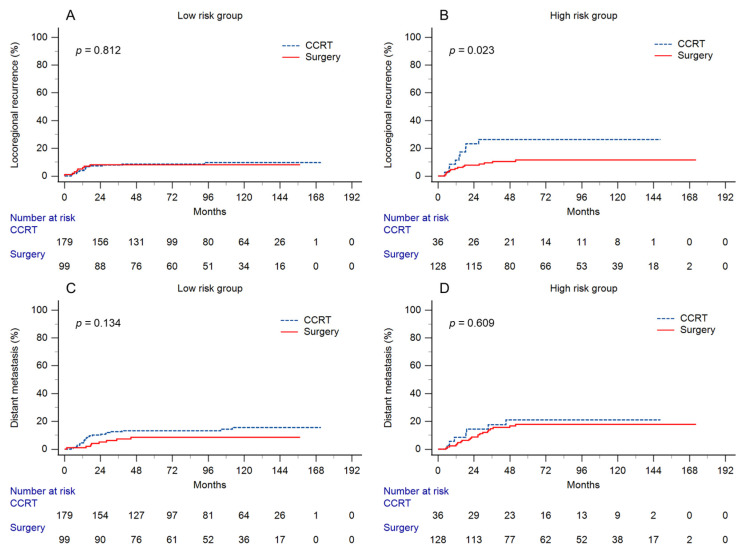
Cumulative incidence of (**A**) locoregional recurrence and (**C**) distant metastasis in the low-risk group and (**B**) locoregional recurrence and (**D**) distant metastasis in the high-risk group by two treatment modalities. Patients treated with primary surgery had a lower locoregional recurrence rate than those treated with concurrent chemoradiation in the high-risk group.

In the entire cohort, the 5-year OS in the surgery and CCRT groups was 84.4% vs. 83.3% (*p* = 0.352), respectively, and the RFS, LRR, and DM were 79% vs. 78.7% (*p* = 0.54), 10% vs. 11.5% (*p* = 0.496), and 13.7% vs. 14.5% (*p* = 0.467), respectively. After risk stratification, the 5-year OS rates of the patients undergoing surgery and CCRT were 85.9% and 85.4%, respectively (*p* = 0.315), in the low-risk group. In the high-risk group, the 5-year OS was 83.2% and 73.3% in the patients undergoing surgery and CCRT, respectively (*p* = 0.164). Regarding RFS, there was no difference in the low-risk group between the patients receiving surgery and those receiving CCRT, with 5-year RFS rates of 83.6% vs. 82.5%, respectively (*p* = 0.558). However, in the high-risk group, the 5-year RFS was significantly higher in the patients undergoing surgery than in those undergoing CCRT (75.2% vs. 59.6%, *p* = 0.036). The comparison of OS and RFS between surgery and CCRT in each group is shown in Figure 2. In view of the recurrence pattern, the 5-year incidence of LRR in the low-risk group was similar between the treatment modalities of surgery and CCRT (8.1% vs. 8.6%, *p* = 0.812). Meanwhile, there was a significantly lower incidence of LRR in the patients receiving surgery than in those receiving CCRT in the high-risk group (11.6% vs. 26.3%, *p* = 0.023). There was no difference in the 5-year incidence of DM between surgery and CCRT in either the low-risk (8.5 vs. 13.2%, *p* = 0.134) or high-risk (17.8% vs. 21%, *p* = 0.609) group. The comparison of recurrence patterns is shown in Figure 3. Moreover, in the low-risk group, the 5-year RFS of patients with and without completion of brachytherapy were 67.9% and 83.8%, respectively (*p* = 0.270). In the high-risk group, the 5-year RFS of patients with and without completion of brachytherapy were 25% and 64%, respectively (*p* = 0.011).

In the multivariate analysis, tumor differentiation, hemoglobin, FIGO stage, and treatment modality were not significantly associated with OS or the incidence of DM in either the high-risk or low-risk group. Regarding RFS and LRR, multivariate analysis revealed that surgery was associated with significantly higher RFS (HR: 0.459, 95% CI: 0.229–0.919, *p* = 0.028) and a lower incidence of LRR (HR: 0.317, 95% CI: 0.123–0.816, *p* = 0.017) in the high-risk group but not in the low-risk group. The other clinical factors, including histology differentiation, hemoglobin, and FIGO stage, were not associated with either RFS or LRR. Table 2 shows multivariate analyses for OS, RFS, LRR, and DM. Moreover, multivariate analysis was also performed to evaluate the impact of neoadjuvant chemotherapy on oncologic outcomes in the entire surgery group (Table 3). Neoadjuvant chemotherapy did not significantly affect oncologic outcomes, including OS (HR: 0.584, 95% CI: 0.174–1.955, *p* = 0.383), RFS (HR: 0.413, 95% CI: 0.126–1.355, *p* = 0.145), LRR (HR: 0.283, 95% CI:0.037–2.162, *p* = 0.224), and DM (HR:0.384, 95% CI:0.089–1.646, *p* = 0.197).

## 4. Discussion

For early bulky cervical tumors, there is a paucity of studies clearly defining the low-risk or high-risk group. We defined the low- and high-risk groups based on the viewpoint of possible different treatment responses between surgery and radiotherapy. Previous studies [10,11,12] revealed that pathology-adenocarcinoma/adenosquamous cell carcinoma and high tumor marker CEA influence the clinical outcome in patients receiving radiation due to the radioresistant nature of tumors. On a molecular basis, radioresistant adenocarcinoma of the cervix has been reported to have an increased frequency of non-homologous end joining (NHEJ) proteins DNA–PKcs, Ku70, and Ku86 repairing DNA lesions [13]. Moreover, clinically, more AC/ASC patients had residual indurations with incomplete tumor regression over the cervix after RT than those SCC patients (40% vs. 22%) [14]. In terms of tumor marker, high CEA level in SCC patients receiving CCRT is associated with local failure and worse disease-free survival [12]. We take a CEA level > 10 ng/mL as the cutoff value due to the clinical prediction of significantly poor CCRT outcomes [10,12]. Other prognostic factors such as low hemoglobin level and poor differentiation have not been reported to potentially become the criteria of classification to show differences between treatment modalities. Therefore, under classification by these high-risk features, we could expect to see the discrepancy between the surgery and CCRT and guide treatment selection.

Although the optimal treatment modality for early bulky tumors remains controversial (treatment outcome listed in Table 4), for patients with high-risk features of poor RT response, surgery should be considered the first priority of initial treatment. In the present study, the histology of AC/ASC and tumor marker CEA ≥ 10 ng/mL in patients with SCC were chosen as the risk factors to stratify the patients into groups. Previous studies reported that patients with these factors have a worse prognosis when receiving radiotherapy [10,11,12]. For early bulky tumors with these high-risk features, we found that radiation combined with concurrent chemotherapy led to approximately 15% more local failure and less RFS despite similar survival compared with primary surgery. Methods for potential improvement of outcome with radiotherapy included three-dimensional (3D) image-guided brachytherapy, increased radiobiological dose, and stereotactic body radiation therapy (SBRT) as a boost treatment for patients unable to undergo brachytherapy. The advantages of 3D image (MRI or CT)-guided brachytherapy include improved tumor coverage (especially for bulky disease), decreased dose to critical organs, and confirmation of applicator placement [15]. On the other hand, the LRR and RFS of CCRT were comparable to those of RH in patients without risk factors. Thus, the treatment choice should be based on patient group selection.

For patients undergoing radiotherapy, the poor radiosensitivity of AC/ASC contributes to a worse prognosis [11,16,17]. A previous study reported a poorer complete response rate (CR) and longer median time to achieve CR in patients with AC/ASC than in those with SCC from definitive RT or CCRT [16,18]. In patients with early bulky tumors, a higher percentage of AC/ASC also had an impact on the treatment outcome from radiotherapy. In one prospective trial, Landoni et al. compared the outcome of surgery and RT alone in 109 women with IB2 and IIA2 with initiation in 1997 and follow-up for 20 years [3,19]. There was no significant difference between RH and RT in OS and RFS, whereas patients with adenocarcinoma had worse OS after definitive RT. Huang et al. [11] showed that patients with IB-IIA bulky cervical tumors receiving definitive RT or CRT had a worse survival of 50% and disease-free survival of 38%. The pathology in all patients was AC/ASC. Zivanovic et al. [20] reported a worse prognosis, with only 50% disease-free survival in FIGO stage IB2 cervical cancer patients receiving definitive RT or CRT. The percentage of AC/ASC accounted for more than 30% in that cohort study, higher than those from other retrospective studies [21,22,23,24] (ranging from 3% to 12%). In the present study, patients in the high-risk group receiving RT had 73% OS and 60% DFS. In our RT group, all high-risk patients with AC/ASC had completed CCRT. The addition of concurrent chemotherapy might contribute to the OS benefit, which is consistent with the findings of a previous study [23] showing that CCRT was better than definitive RT. In contrast to primary RT/CCRT, surgery was a more effective treatment option for bulky IB-IIA tumors. In patients with AC/ASC, Huang et al. [11,25] showed that radical hysterectomy resulted in better 5-year local relapse-free survival than RT/CCRT for IB-IIA bulky cervical tumors (91% vs. 48%, respectively). In our cohort, despite CCRT instead of RT, the RFS and local control of primary surgery were still superior to those of radiation-first treatment. This indicated that the radioresistant nature of AC/ASC might be overcome by surgery. Therefore, for these bulky AC/ASC tumors, surgery should be considered first.

The serum CEA level was less commonly elevated in patients with SCC (22%) than in those with endocervical AC (40%) [26]. However, elevated pretreatment CEA predicts poor prognosis in SCC of the cervix [10,12]. In patients with advanced-stage cervical cancer undergoing CCRT, Chen et al. [10] reported that pretreatment CEA > 10 ng/mL portends worse cancer-specific survival and disease-free survival. CEA levels have been found to have a stronger correlation with radioresistance than SCC-Ag levels [14]. A previous study indicated that cervical tumors producing more CEA are analogous to radioresistant adenocarcinoma [14]. In our cohort, more patients with ASC/AC underwent surgery than CCRT because most physicians regarded these histological subtypes as associated with a slow and poor response to RT. Meanwhile, more patients with SCC but CEA ≥ 10 ng/mL underwent CCRT than surgery because SCC was perceived to be more relatively radiosensitive than AC/ASC. However, after separating the patients with AC/ASC from SCC but CEA ≥ 10 ng/mL, we found that the 5-year RFS and LRR for patients with SCC but CEA ≥ 10 ng/mL undergoing CCRT were 62.5% and 25%, respectively; the corresponding outcomes for patients with AC/ASC were 57.8% and 27.4%. This indicated that patients with SCC but a pretreatment CEA level ≥ 10 ng/mL had not only lower RFS but also high local failure after CCRT, similar to those with AC/ASC who underwent CCRT. This result was compatible with the data from Huang et al. [12], showing that a high CEA level was associated with local failure (hazard ratio: 2.5) and DFS (HR: 2.73). Therefore, radiation-based treatment might not be more effective than surgery for patients with CEA levels ≥ 10 ng/mL.

Based on our study results, although SCC was relatively radiosensitive, patients with high CEA levels had poor RT responses. It could be hypothesized that CEA-producing tumor cells have a poor response to CCRT similar to AC/ASC and coexist in patients with SCC. Previous studies reported the possibility of the simultaneous presence of SCC and AC in the uterine cervix [27,28,29]. SCC originates at the transformation zone, and AC arises from the cylinder mucosa of the endocervix or endocervical glands. For bulky tumors with the coexistence of these two types of pathology, SCC was predominantly sampled from the exocervix, whereas AC can be localized deep in the endocervical canal and easily missed during biopsy because of difficulty in entering the internal cervical os. The elevated CEA level might originate from the presence of an AC component with difficulty in tissue proof. Therefore, for patients with SCC but high CEA levels, CCRT might not completely eradicate the radioresistant tumor cells behind radiosensitive SCC, and the treatment outcome in our study was compatible with this finding.

**Table 4:** Literature review of treatment outcomes for early-staged bulky cervical cancer.

Author/Year	N	Stage (FIGO 2009)	5-Year OS (%)	5-Year DFS (%)	LRR (%) ^a^	DM (%) ^a^
**Surgery (RH)**						
Landoni 1997 [3]	55	IB2/IIA2	70	63	20	15
Yessaian 2004 [30]	58	IB2	62	na	29	5
Havrilesky 2004 [31]	72	IB2	72	63	24	7
Zivanovic 2008 [20]	27	IB2	72 ^b^	52 ^b^	37	7
Kim 2011 [21]	28	IB2	82	na	29	7
Huang 2012 [25]	60	IB2/IIA2	na	SCC: 74	na	na
				AC/ASC: 62		
Rungruang 2012 [6]	401	IB2	83 ^c^	87 ^c^	na	na
Park 2012 [22]	147	IB2/IIA2	78	77	13	8
Alleyne-Mike 2013 [23]	25	IB2	88	na	8	4
Derks 2016 [32]	129	IB2/IIA2	83	82	12	14
Bradbury 2015 [33]	67	IB2	75	67	12	19
Liu 2020 [24]	235	IB2/IIA2	82	73	na	na
Li 2022 [34]	280	IB3 (FIGO 2018)	94	93	na	na
Present study	Entire cohort: 227	IB2/IIA2	84	79	10	14
	Low risk: 99	IB2/IIA2	86	84	8	9
	High risk: 128	IB2/IIA2	83	75	12	18
RT/CCRT						
Landoni 1997 [3]	54	IB2/IIA2 (RT)	72	57	30	13
Chang 2000 [35]	52	IB2/IIA2 (RT)	61	50	15	15
Keys 2003 [36]	124	IB2 (RT)	62	53	27	15
Zivanovic 2008 [20]	20	IB2 (RT/CCRT)	55	55	40	20
Kim 2010 [21]	35	IB2 (RT/CCRT)	76	na	14	9
Huang 2011 [11]	34	IB2 (RT/CCRT)	50	38	na	na
Rungruang 2012 [6]	369	IB2	63 ^c^	73 ^c^	na	na
Park 2012 [22]	68	IB2/IIA2 (CCRT)	67	66	19	10
Alleyne-Mike 2013 [23]	53	IB2 (RT/CCRT)	All:63	na	11	15
			RT/0-3 CHT ^d^:39			
			RT/4-6 CHT ^e^: 79			
Bradbury 2015 [33]	25	IB2 (RT/CCRT)	60	56	28	16
Liu 2020 [24]	235	IB2/IIA2 (CRT) ^f^	73	73	na	na
Li 2022 [34]	108	IB3 (FIGO 2018) (CRT) ^f^	76	69	na	na
Present study	Entire cohort:215	IB2/IIA2 (CCRT)	83	79	12	15
	Low risk: 179	IB2/IIA2 (CCRT)	85	83	9	13
	High risk: 36	IB2/IIA2 (CCRT)	73	60	26	21

OS = overall survival, DFS = disease-free survival, LRR = locoregional recurrence, DM = distant metastases, na = not assessable, ^a^ the patients with both distant and locoregional recurrence were included, ^b^ 3-year OS and DFS, ^c^ Extracted from survival curve ^d^ 0–3 cycles of concurrent chemotherapy, ^e^ 4–6 cycles of concurrent chemotherapy, ^f^ concurrent chemotherapy was not mentioned.

There are several limitations in the present study. The database lacks information regarding the patients’ comorbidities and baseline performance status. Patients receiving initial radiotherapy might have a lower performance status, which has a greater impact on overall survival than disease-free survival. Our study indicated that overall survival is not worse in patients undergoing radiation therapy first. In addition, although the tumor size and nodal status were assessed clinically mainly from either pretreatment CT or MR image, the imaging modalities (CT or MRI) were not recorded. For patients with positive nodal disease, there is also no information about the exact location (pelvic or para-aortic region) of metastatic lymph nodes, causing ambiguity of 2018 staging (IIICI or IIIC2). Moreover, a central pathologic review was not performed due to the dearth of pathologic prognostic factors, including depth of tumor invasion and lymphovascular space invasion. The presence of human papillomavirus (HPV) was also not recorded in our database. Finally, the cancer registry database of our study lacks information regarding treatment-related toxicity. The morbidities of surgery consist of bladder dysfunction, bleeding, and chronic lymphedema [37,38,39], while radiation causes cystitis and possible bowel complications (diarrhea, obstruction, and perforation) [40,41]. Moreover, patients undergoing CCRT experience vaginal stenosis, which may negatively impact their quality of life [42,43]. The difference in treatment-related toxicity may influence treatment selection. Further research on treatment toxicity was warranted.

## 5. Conclusions

The primary strength of our study is the large number of patients with early-stage bulky cervical cancer who were examined. Our study found that in early-stage cervical cancer patients with large tumors and high-risk features (1. SCC histology with CEA level ≥ 10 ng/mL; 2. AC/ASC histology), primary radical surgery with or without adjuvant radiation was suggested for better RFS and local control. In patients without high-risk features, both radical surgery with or without adjuvant radiation and primary CCRT offer effective treatment. Further randomized studies are needed to validate the results.

## Figures and Tables

**Table 1:** Patient characteristics (*n* = 442).

Bulky Tumor	Low Risk (*n* = 278)			High Risk (*n* = 164)		
Treatment Modality	CCRT	Surgery	*p*	CCRT	Surgery	*p*
Patient number	179	99		36	128	
Age (yr)						
≤45	54 (30.2)	34 (34.3)	0.474	15 (41.7)	61 (47.7)	0.524
>45	125 (69.8)	65 (65.7)		21 (58.3)	67 (52.3)	
2009 FIGO stage						
IB2	121 (67.6)	85 (85.9)	0.001	26 (72.2)	113 (88.3)	0.018
IIA2	58 (32.4)	14 (14.1)		10 (27.8)	15 (11.7)	
SCC (ng/mL)	7.8 ± 1.0	5.8 ± 1.0	0.218	4.8 ± 1.4	2.9 ± 1.0	0.299
CEA (ng/mL)	2.6 ± 0.2	2.8 ± 0.2	0.528	36.1 ± 13.4	41.7 ± 27.0	0.908
Hb (g/dL)	11.9 ± 0.1	12.2 ± 0.2	0.198	11.9 ± 0.3	12.2 ± 0.2	0.332

Values are presented as number (%) or mean ± standard error of the mean (SEM).

**Table 2:** Multivariate analyses of survival and recurrence in each risk group.

Low-Risk Group																
	Overall Survival				Recurrence-Free Survival				Locoregional Recurrence				Distant Metastasis			
	*p*-Value	HR	95.0% CI for HR		*p*-Value	HR	95.0% CI for HR		*p*-Value	HR	95.0% CI for HR		*p*-Value	HR	95.0% CI for HR	
Surgery	0.530	0.805	0.409	1.585	0.987	0.995	0.533	1.858	0.587	1.285	0.587	1.285	0.236	0.607	0.266	1.387
IIA2	0.951	1.021	0.520	2.006	0.656	1.150	0.621	2.129	0.750	1.156	0.750	1.156	0.823	1.090	0.513	2.316
HB < 12	0.341	1.384	0.709	2.700	0.520	1.225	0.660	2.276	0.779	0.872	0.779	0.872	0.654	1.188	0.560	2.518
Poor differentiation	0.448	1.303	0.657	2.585	0.225	1.507	0.777	2.921	0.158	2.179	0.158	2.179	0.159	1.807	0.794	4.114
**High-Risk Group**																
	**Overall Survival**				**Recurrence-Free Survival**				**Locoregional Recurrence**				**Distant Metastasis**			
	***p*-Value**	**HR**	**95.0% CI for HR**		***p*-Value**	**HR**	**95.0% CI for HR**		***p*-Value**	**HR**	**95.0% CI for HR**		***p*-Value**	**HR**	**95.0% CI for HR**	
Surgery	0.121	0.533	0.240	1.181	0.028	0.459	0.229	0.919	0.017	0.317	0.123	0.816	0.595	0.777	0.306	1.973
IIA2	0.893	1.064	0.429	2.636	0.737	1.147	0.515	2.557	0.528	0.664	0.187	2.365	0.497	1.382	0.543	3.515
HB < 12	0.375	1.391	0.670	2.888	0.396	0.732	0.356	1.505	0.585	0.769	0.300	1.973	0.728	1.161	0.500	2.698
Poor differentiation	0.485	0.752	0.337	1.675	0.362	1.352	0.707	2.583	0.623	0.774	0.279	2.147	0.514	1.304	0.587	2.895

**Table 3:** Multivariate analyses of survival and recurrence in the surgery group.

Surgery Group																
	Overall Survival				Recurrence-Free Survival				Locoregional Recurrence				Distant Metastasis			
	*p*-Value	HR	95.0% CI for HR		*p*-Value	HR	95.0% CI for HR		*p*-Value	HR	95.0% CI for HR		*p*-Value	HR	95.0% CI for HR	
Neoadjuvant chemotherapy (NACT)	0.383	0.584	0.174	1.955	0.145	0.413	0.126	1.355	0.224	0.283	0.037	2.162	0.197	0.384	0.089	1.646
IIA2	0.417	0.608	0.183	2.019	0.41	0.645	0.228	1.83	0.975	0.62	0.22	2.02	0.821	1.133	0.383	3.348
HB < 12	0.452	1.326	0.636	2.765	0.813	1.083	0.56	2.097	0.495	1.443	0.504	4.133	0.728	1.15	0.522	2.535
Poor differentiation	0.789	0.899	0.413	1.957	0.872	1.057	0.54	2.07	0.067	2.386	0.942	6.045	0.849	0.922	0.4	2.128

## Data Availability

The data presented in this study are available in this article.
